# The right posterior inferior frontal gyrus contributes to phonological word decisions in the healthy brain: Evidence from dual-site TMS

**DOI:** 10.1016/j.neuropsychologia.2010.06.032

**Published:** 2010-08

**Authors:** Gesa Hartwigsen, Cathy J. Price, Annette Baumgaertner, Gesine Geiss, Maria Koehnke, Stephan Ulmer, Hartwig R. Siebner

**Affiliations:** a Department of Neurology, Christian-Albrechts-University, Kiel, Germany; b NeuroImage-Nord, Hamburg-Kiel-Lübeck, Germany; c Wellcome Trust Centre for Neuroimaging, University College London, United Kingdom; d Department of Speech Language Pathology, Hochschule Fresenius Hamburg, Germany; e Institute of Neuroradiology, University Hospital of Schleswig-Holstein, Kiel, Germany; f Interventional and Diagnostic Neuroradiolgy, University Hospital Basel, Switzerland; g Danish Research Centre for Magnetic Resonance, Copenhagen University Hospital Hvidovre, Copenhagen, Denmark

**Keywords:** Language, Human brain, Hemisphere, Transcranial magnetic stimulation, Compensation, Functional magnetic resonance imaging

## Abstract

There is consensus that the left hemisphere plays a dominant role in language processing, but functional imaging studies have shown that the right as well as the left posterior inferior frontal gyri (pIFG) are activated when healthy right-handed individuals make phonological word decisions. Here we used online transcranial magnetic stimulation (TMS) to examine the functional relevance of the right pIFG for auditory and visual phonological decisions. Healthy right-handed individuals made phonological or semantic word judgements on the same set of auditorily and visually presented words while they received stereotactically guided TMS over the left, right or bilateral pIFG (*n* = 14) or the anterior left, right or bilateral IFG (*n* = 14). TMS started 100 ms after word onset and consisted of four stimuli given at a rate of 10 Hz and intensity of 90% of active motor threshold. Compared to TMS of aIFG, TMS of pIFG impaired reaction times and accuracy of phonological but not semantic decisions for visually and auditorily presented words. TMS over left, right or bilateral pIFG disrupted phonological processing to a similar degree. In a follow-up experiment, the intensity threshold for delaying phonological judgements was identical for unilateral TMS of left and right pIFG. These findings indicate that an intact function of right pIFG is necessary for accurate and efficient phonological decisions in the healthy brain with no evidence that the left and right pIFG can compensate for one another during online TMS. Our findings motivate detailed studies of phonological processing in patients with acute and chronic damage of the right pIFG.

## 1 Introduction

Functional imaging studies have shown that right as well as left posterior inferior frontal gyri (pIFG) are activated when healthy right-handed subjects perform phonological tasks (Chee, O’Craven, Bergida, Rosen, & Savoy, 1999; Devlin, Matthews, & Rushworth, 2003; Poldrack et al., 1999; Shibahara, 2004; Tremblay, Monetta, & Joanette, 2004). This bilateral pIFG activation pattern is surprising given that lesion studies emphasize that phonological processing is more impaired after left than right inferior frontal damage (e.g. Dewarrat et al., 2009; Wilde, 2009; Winhuisen et al., 2007) and theoretical models of language focus on the importance of the left rather than right hemisphere (e.g. Shalom & Poeppel, 2008). Transcranial magnetic stimulation (TMS) studies of phonological processing in healthy right-handed subjects have also focused on the functional relevance of left but not right pIFG (e.g. Gough, Nobre, & Devlin, 2005; Nixon, Lazarova, Hodinott-Hill, Gough, & Passingham, 2004; Romero, Walsh, & Papagno, 2006). These studies demonstrated that left pIFG is more involved in phonological than semantic judgements on written words but they did not investigate the role of pIFG in the right hemisphere. To address the discrepancy between functional imaging and lesion studies, the present study was designed to examine how “online” TMS (i.e. TMS during a task) over the left and right pIFG influences phonological word processing in healthy subjects. We used the neurodisruptive effect of TMS to distinguish between three alternative explanations for right pIFG activation with phonologic processing.

Hypothesis 1 Right pIFG contributes to the speed and efficiency of phonological decisions. Consequently, right pIFG lesions have a subtle effect that might be missed unless reaction times were measured. In this case, we expect a significant effect of right pIFG TMS on reaction times but not error rates in the healthy brain.

Hypothesis 2 Right pIFG is necessary for accurate and efficient phonological decisions in the healthy brain but following right pIFG lesions, the function of right pIFG can be supported by alternative brain regions. Consequently, right pIFG lesions may temporarily impair phonological decision performance in the acute phase after brain damage but this lesion effect will not be apparent after functional reorganisation. In this case, we expect a significant effect of right IFG TMS on both the reaction times and accuracy of phonological decisions in the healthy brain.

Hypothesis 3 Right pIFG is not necessary for accurate and efficient phonological decisions but is activated in fMRI studies of the healthy brain because it is involved in task-related activation that is incidental to performance (i.e. redundant processing, Price & Friston, 2002). In this case, neither right pIFG lesions nor right pIFG TMS will influence phonological decision performance.

Our study extends previous online TMS studies of phonological processing in three ways. First, we investigated the effect of TMS to the right pIFG. Second, we compared unilateral TMS over the right pIFG to unilateral TMS to the left pIFG and dual-site TMS over left and right pIFG simultaneously. This manipulation allowed us to test whether impaired unilateral pIFG function was supported by the contralateral hemisphere. If so, then the effect of dual-site TMS to both the left and right pIFG should be greater than the effect of TMS to either the left or right pIFG alone (Price & Friston, 2002). Third, we compared the effect of TMS on phonological decisions to words presented in the auditory as well as visual modality, whereas previous studies investigated the effect of online TMS to left pIFG with visually presented words only (Gough et al., 2005; Nixon et al., 2004; Romero et al., 2006). This enabled us to assess whether the expected TMS effects were dependent or independent of stimulus modality.

To test the functional specificity of our effects, we also investigated how online TMS to the same pIFG sites affected semantic decisions on the same sets of stimuli. Finally, to test the regional specificity of any observed effects, we investigated how phonological decisions were affected by TMS over anterior inferior frontal gyri (aIFG). Functional imaging studies have demonstrated a functional-anatomic subdivision within the IFG with more anterior regions being preferentially engaged in semantic processing and more posterior regions in phonological processing (e.g. Burton, 2001; Fiez, 1997; Gold, Balota, Kirchhoff, & Buckner, 2005; Poldrack et al., 1999; Wise, 2003). On the basis of a recent TMS study by Gough et al. (2005), we expected that phonological but not semantic judgements would be impaired with TMS applied to the pIFG but not aIFG.

## 2 Materials and methods

### 2.1 Subjects

28 right-handed native German speakers with no history of neurological disorders or head injury were randomly assigned to the pIFG TMS group (*n* = 14, 8 females, 20–28 years old, mean age 24.01 ± 2.38) or the aIFG TMS group (*n* = 14, 7 females, 20–30 years old, mean age 23.85 ± 2.53). We also included a control group of another 7 healthy subjects (3 females, 21–27 years old, mean age 24.03 ± 2.32) receiving only sham TMS. Written informed consent was obtained before the experiment. Handedness was tested with the German version of the Edinburgh Handedness Inventory (Oldfield, 1971). All subjects had normal or corrected-to-normal visual acuity and were naive to TMS. The study was performed according to the guidelines of the Declaration of Helsinki and approved by the Ethics Committee of the Medical Faculty of the Christian-Albrechts-University of Kiel.

### 2.2 Experimental design

**Fig. 1:**
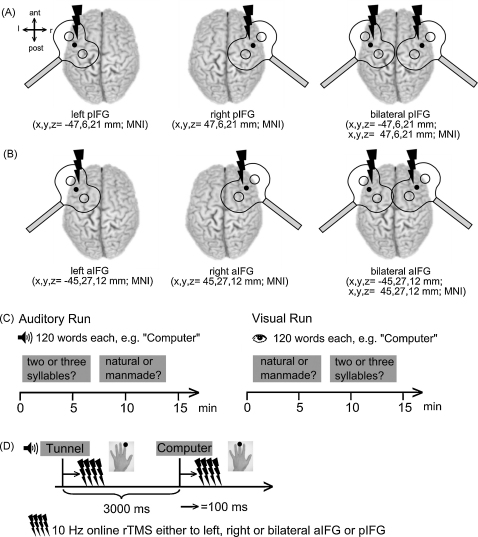
Experimental design. (A) and (B) Stimulation sites over the left, right and bilateral pIFG and aIFG; respectively. Mean MNI-coordinates were obtained from previous studies (Devlin et al., 2003; Gitelman et al., 2005; Gough et al., 2005; McDermott et al., 2003). ant = anterior, post = posterior, l = left, r = right. (C) Auditory and visual run of the two blocked tasks. (D) Single trial: each trial had a duration of 3000 ms. A 4-pulse train of 10 Hz online TMS was applied 100 ms after word onset over left, right or bilateral aIFG or pIFG. Subjects responded with their left index or middle finger. ms = milliseconds; min = minutes.

The experiment had a 2 × 2 × 3 × 2 design with two different tasks (phonological and semantic) in two modalities (auditory and visual) and three TMS sites (left, right, and bilateral stimulation) for two groups receiving TMS either over the pIFG or aIFG, respectively (Fig. 1A and B). An identical set of 120 stimuli was presented in each of the two tasks in both the auditory and visual modalities. This resulted in four repetitions of the same words with the effect of repetition controlled across tasks. In order to keep the repetition of identical stimuli per subject at a minimum (i.e. four) we decided against treating the pIFG vs. aIFG TMS conditions as within subject factor and thus included two different groups. The factorial design enabled us to test for task, site and group specific modality-independent effects while controlling for stimulus and repetition effects. The control group received only sham TMS to test whether the different tasks yielded comparable results with respect to reaction times (RT) and error rates (ER) without the influences of real TMS (supplementary data). In all other aspects the experimental design for the control group was comparable to the main experiment.

### 2.3 Tasks

Subjects performed two different tasks in the visual and the auditory modality on the same stimuli in both tasks. In the phonological task, subjects categorized the items as having two or three syllables. The semantic task consisted of deciding whether a word represented a natural or manmade item. Tasks were blocked to ensure a constant cognitive set. Subjects were instructed to respond as quickly and as accurately as possible by pressing a button on a response pad with their left middle or index finger (Fig. 1D).

### 2.4 Stimuli

60 two-syllable and 60 three-syllable German words were used for stimulus presentation. Only highly frequent, unambiguous nouns from the CELEX lexical database for German (Centre for Lexical Information, Max Planck Institute for Psycholinguistics, The Netherlands) were selected. No compound nouns, hypernyms or foreign words were included. Thirty German native speakers (15 females, age 24–47, mean age 29.0) independently categorized each item as either manmade or natural, rated each item's imageability on a four-point scale, ranging from 1 (concrete) to 4 (abstract), and provided the number of syllables for each item. These subjects were not included in the present study.

Words were only included if (i) at least 29 out of 30 pilot subjects correctly classified them as being either manmade or natural, (ii) they received an average imageability rating of <1.6, and (iii) they reached >90% agreement on the intended syllable count. Since more two-syllable than three-syllable words passed the above validation criteria, we were able to select the two-syllable nouns that most closely matched the three-syllable words in terms of their imageability ratings and number of letters (to the degree possible). In total, 60 two-syllable nouns and 60 three-syllable nouns were selected. All words represented natural or manmade items (50% each).

Auditory versions of the words were recorded by a professional female speaker and had an average duration of 0.74 s (range: 0.52–1.02 s, two-syllable words) and 0.87 s (range: 0.66–1.12 s, three-syllable words), respectively.

### 2.5 Procedure

As a prerequisite for neuronavigated TMS, all subjects underwent MR imaging using a MPRage sequence in sagittal orientation (slice thickness 1 mm; in plane resolution 1 mm × 1 mm; TE/TR = 3.78/8.25 ms). After stereotactic coregistration and determination of the individual motor threshold with transcranial magnetic stimulation (see below), the experiment was explained and subjects performed a training session with three trials per task. None of the stimuli used in that session were repeated in the main experiment. During the practice session, sound volume was individually adjusted for each subject (with a range of 99–105 decibel [dB(A)]). Auditory stimuli were presented via in-ear headphones equipped with earplugs to shield the subject from the TMS induced noise. For further shielding, a foam cushion was fixed around the subject's head above the ears during the whole procedure. During volume adjustment, TMS coils were charged closely above the subject's head to induce noise that was comparable to the experimental session. Visual stimuli were presented in the center of a computer monitor in front of the subject (19 in. flat-screen monitor, resolution: 1280 × 1024 pixels, distance from the subject: 70 cm). The font size for presentation was set to 86 pt.

After the training session, the two TMS coils were stereotactically positioned over the left and right pIFG or aIFG (Fig. 1A and B) and remained fixed during the experiments. Subjects received three test bursts of 10 Hz TMS over left, right and bilateral pIFG or aIFG and judged them on a 4-point scale either as “neutral” (1), “moderate unpleasant” (2), “unpleasant” (3) or “highly unpleasant” (4). The unpleasantness scores were implemented to assess whether TMS was comparable for all stimulation sites (i.e. left, right and bilateral aIFG or pIFG).

The experiment consisted of an auditory and a visual run for each subject (Fig. 1C). The order of runs was counterbalanced across subjects. During each run the two blocked tasks were randomly presented and each task started with a verbal or written instruction of the task and consisted of 120 trials for each condition, with a trial-duration of 3 s (Fig. 1D). Presentation of visual words was matched to the mean duration of the auditory stimuli (range = 0.74–0.87 s) and followed by a fixation cross to complete the 3-s trial. During the auditory run, the fixation cross stayed on the screen for the whole experiment. Having completed all four conditions, subjects again rated the unpleasantness of the three TMS sites.

The control group underwent exactly the same experimental procedure including MR scan, stereotactically guided coil positioning and unpleasantness ratings. For each experimental condition, mean reaction times (RT) and error rates (ER) were calculated. Stimulus presentation and response recording was obtained using E-PRIME software (Psychology Software Tools Inc., Pittsburgh, PA, USA; version 1.1).

### 2.6 Transcranial magnetic stimulation

We used neuronavigated TMS (TMS-Navigator, Localite, Sankt Augustin, Germany) based on the coregistered individual T1-weighted MR image to navigate the TMS coils and maintain their exact location and orientation throughout the experimental sessions. Neuronavigated TMS was performed by using the mean MNI-coordinates for left pIFG across four recent studies comparing visual presented words in a word comprehension task (−47 6 21; Devlin et al., 2003; Gitelman, Nobre, Sonty, Parrish, & Mesulam, 2005; Gough et al., 2005; McDermott, Petersen, Watson, & Ojemann, 2003 see Fig. 1A). Stereotactic coordinates for left aIFG (*x*,*y*,*z* = −45, 27, and 12 mm) were obtained from group activation data from a previous fMRI study which used the same experimental paradigm in an independent sample of subjects (A. Baumgaertner, G. Hartwigsen, and H.S. Siebner, unpublished data). Thus, we used the modality-independent comparison between the semantic and phonological task in our previous fMRI study (Fig. 1B). For right hemisphere TMS we used the contralateral homologue areas. Using these stereotactic coordinates, the individual stimulation sites were determined by calculating the inverse of the normalisation transformation and transforming the coordinates from standard to “individual” space for each subject. A recently developed algorithm (http://r-forge.r-project.org/projects/rniftilib/) calculated the shortest distance from the target coordinate in the brain to the surface for each subject. The TMS coils were placed over these “entry-coordinates” on the surface of the head.

The coils were placed tangentially on the head with the handle pointing at 45° to the sagittal plane, with the second phase of the biphasic pulse inducing a posterior to anterior current flow (Fig. 1A and B). Stimulation intensity was set to 90% of individual active motor threshold (AMT). AMT was defined as the lowest stimulus intensity producing an MEP of approximately 150–200 μV in the tonically active first dorsal interosseus muscle (20% of maximum contraction). Mean stimulation intensities were 28.07 ± 5.64% and 28.96 ± 3.19 total stimulator output for pIFG and aIFG, respectively. Figure-of-eight shaped coils (double 90 mm; coil type Q.C., Mag and More GmbH, Munich, Germany) and P-Stim 160 stimulators (Mag and More GmbH, Munich, Germany) were used in all TMS conditions.

During each experimental trial, a four-pulse train of stereotactically guided 10 Hz TMS was applied over left, right or bilateral pIFG or aIFG 100 ms after word onset (Fig. 1D).

Trials with left, right and bilateral TMS (40 each) were pseudorandomly intermingled. The overall application of TMS was well within safety limits (Wassermann, 1998). In the control group receiving sham TMS, an additional coil was placed in an angle of 90° over each coil. Stimulation intensity of these coils was set 15% higher to create a comparable acoustic stimulus without stimulating the brain. Trials with left, right and bilateral sham TMS (40 each) were pseudorandomly intermingled.

### 2.7 Data analysis

Reaction times for trials with correct responses were examined with a four-way repeated measures ANOVA. The 2 × 2 × 3 × 2 ANOVA model included the within-subject factors task (phonological vs. semantic), modality (auditory vs. visual stimulus presentation) and TMS site (left, right, or bilateral) and a between-subjects factor group (TMS over pIFG vs. TMS over aIFG).

The Greenhouse-Geisser correction was used to correct for non-sphericity when appropriate. Conditional on significant *F*-values, post hoc paired *t*-tests were used to further characterize differences among conditions within groups. Between group differences were examined using independent samples *t*-tests, an α-level of 0.05 was considered significant for all comparisons. All reported *p*-values are two-tailed.

Results from the control group (sham TMS) were analysed separately to test whether the different tasks yielded comparable results with respect to reaction times (RT) and error rates (ER) without the influences of real TMS. RT for trials with correct responses were examined using a three-way repeated measures ANOVA with the factors task (phonological, semantic), modality (auditory vs. visual) and TMS site (left, right or bilateral sham stimulation).

We used Bonferroni–Holm corrected non-parametric Wilcoxon signed-rank tests and Mann–Whitney *U* tests for statistical analyses of error rates since Kolmogorov–Smirnov tests had indicated that these data were not normally distributed, precluding the use of an ANOVA.

For the comparisons on ER within the control group, no Bonferroni–Holm correction was applied since we wanted to test the null-hypothesis (e.g. no significant differences between the tasks). Unpleasantness ratings were also analysed with non-parametric Wilcoxon signed-rank tests and Mann–Whitney *U* tests without Bonferroni–Holm correction since the null-hypothesis (no significant differences between the three stimulation sites) should be maintained. All statistical analyses were performed with SPSS Software (version 13, Chicago, IL, USA).

## 3 Results

### 3.1 Reaction times

Subjects’ mean reaction times (RT) were examined with a four-way repeated measures ANOVA. The ANOVA model included the factors: task (phonological vs. semantic), modality (auditory vs. visual), TMS site (left, right, bilateral) and group (pIFG vs. aIFG). Table 1 lists mean RT and ER for the phonological and semantic task in the pIFG and aIFG groups.

Overall, RT were longer when subjects made phonological compared to semantic judgements. This was indicated by a main effect of task pooled over the factors TMS site (left, right or bilateral), modality (auditory and visual) and group (pIFG and aIFG) (*F*_1,26_ =  12.94; *p* = 0.001).

There was also a main effect of modality due to longer RT for auditorily than visually presented words across tasks, TMS sites and groups (*F*_1,26_ = 199.76 ; *p* =  0.0001).

**Fig. 2:**
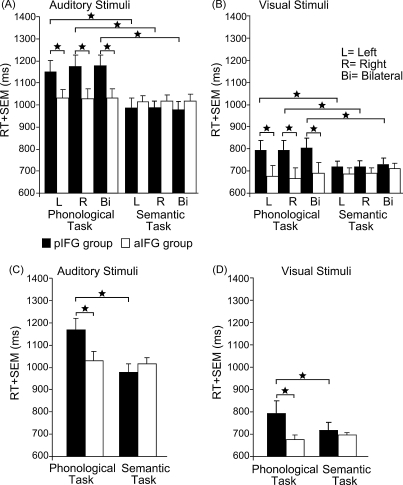
Mean reaction times (RT) for the phonological and semantic task in the aIFG and pIFG group. For illustrating purposes, responses for auditorily and visually presented stimuli are displayed in different panels. The significant two-way interaction between task and group is displayed in all panels. Note that the three different TMS sites (left, right, bilateral) are shown separately in (A) and (B) for illustrating purposes although the two-way interaction was pooled across the factors TMS site and modality. Error bars represent onefold standard error from the mean (SEM); *p < 0.05; two-tailed; ms = milliseconds.

Repeated-measures ANOVA revealed that TMS over pIFG but not aIFG increased RT for the phonological task only (significant task-by-group interaction: *F*_1,26_ = 13.77; *p* = 0.001; Fig. 2).

Accordingly, post hoc paired comparisons indicated increased RT for the phonological compared to the semantic task in the pIFG group (*t*_27_ = 4.82; *p* < 0.001; post hoc *t*-test) but not in the aIFG group (*p* = 0.89). Overall, the pIFG group showed longer RT in the phonological task relative to the aIFG group (*t*_27_ = 2.02; *p* = 0.048; between-group comparison). In contrast, there were no overall differences in mean RT for the semantic task between both groups (*p* = 0.88). The task-specific delay of phonological decisions with TMS to pIFG was independent of the modality as there was no task-by-group-by-modality interaction. The ANOVA showed no main effect or interaction with the factor TMS site, indicating that unilateral TMS of left and right pIFG as well as dual-site TMS of right and left pIFG produced a similar disruption of phonological judgements.

We also found an interaction between task and modality (*F*_1,26_ = 9.38; *p* =  0.005) pooled across the factors group and TMS site. This interaction indicated that the RT difference between phonological and semantic judgements was greater for auditorily presented words (*t*_27_ = 3.51; *p* = 0.002; post hoc *t*-test) than visually presented words (n.s.; *p* = 0.16).

### 3.2 Error rates

**Fig. 3:**
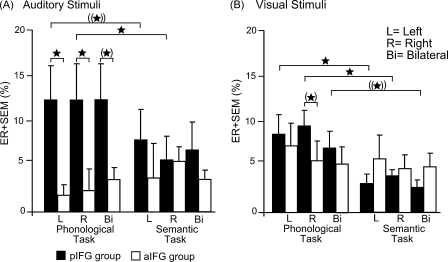
Mean error rates (ER) for the phonological and semantic task in the aIFG and pIFG group. Error bars represent onefold standard error from the mean (SEM); *p < 0.05; two-tailed; (*) did not survive the Bonferroni–Holm correction; ((*))*p* < 0.10 (trend).

Relative to TMS over aIFG, TMS over pIFG resulted in an increase in error rates (ER) when participants made phonological judgements (Fig. 3). The effects that were still significant after Bonferroni–Holm correction for multiple comparisons were as follows: in the auditory modality, phonological errors increased relative to semantic errors when TMS was applied to right pIFG (*Z* = 2.89; *p* = 0.009). There was also a trend for increased error rates for phonological compared to semantic errors in the auditory modality with TMS of left pIFG (*Z* = 1.80; *p* = 0.075; Fig. 3A).

In the visual modality, Wilcoxon signed-rank tests revealed significant increases in phonological errors relative to semantic errors when TMS was given to the left pIFG (*Z* = 2.52; *p* = 0.012) or right pIFG (*Z* = 3.05; *p* = 0.001). A similar trend towards a selective increase in phonological errors was present in the visual modality when TMS was applied to both the left and right pIFG (*Z* = 1.97; *p* = 0.052; Fig. 3B). Neither TMS of pIFG or aIFG caused a significant increase in ER for semantic decisions.

Mann–Whitney *U* tests showed differences in ER between the pIFG and aIFG group: left pIFG TMS was associated with an increase in ER for the auditory phonological task relative to left aIFG (*Z* = 3.18; *p* = 0.001). Right pIFG TMS compared with right aIFG TMS also significantly increased phonological errors in the auditory modality (*Z* = 2.88; *p* = 0.0091). Increased phonological error rates were also present with auditory stimuli when TMS was applied to bilateral pIFG relative to bilateral aIFG (*Z* = 2.05; *p* = 0.042) and with visual stimuli when TMS was given to right pIFG (*Z* = 2.02; *p* = 0.044), however, these comparisons did not survive the Bonferroni–Holm correction. There were no significant differences in semantic errors between the groups (i.e. pIFG and aIFG group) in either modality (Fig. 3).

### 3.3 Follow-up experiment

Our main experiment indicated comparable effects for unilateral TMS over left and right pIFG on phonological processing. In a follow-up experiment, we compared the intensity-dependence of the behavioural “lesion” effect induced by unilateral TMS to the left or right pIFG. We thus wanted to investigate whether the relationship between TMS-intensity and behavioural perturbation for left versus right pIFG were different by constructing intensity-effect-size curves.

More specifically, this experiment enabled us to test if left pIFG TMS disrupted phonological processing at lower intensities than right pIFG TMS.

**Fig. 4:**
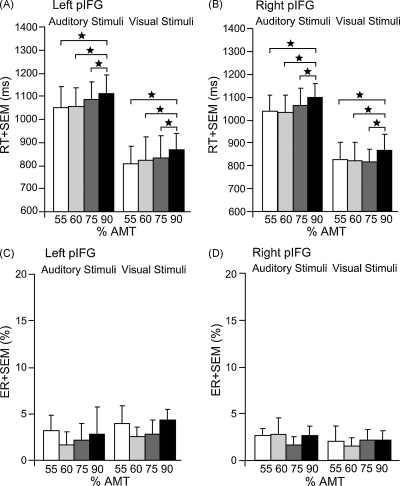
Mean reaction times (RT; A and B) and error rates (ER; C and D) for the phonological task in the follow-up experiment (TMS at different intensities over the left and right pIFG). In (A) and (B), the main effect of intensity on RT is displayed. Note that the two different TMS sites (left and right pIFG) and the two modalities (auditory and visual) are displayed here separately for illustrating purposes although the main effect was pooled across the factors TMS site and modality. For ER, no significant differences were found between the different conditions. Error bars represent onefold standard error from the mean (SEM); *p < 0.05; two-tailed; AMT = active motor threshold.

To this end, 7 subjects (5 females, mean age 22.75 ± 2.54) from both experimental groups (i.e. pIFG vs. aIFG) performed two sessions of the phonological task again while receiving TMS over left (session one) or right pIFG (session two). TMS was applied at four different stimulation intensities with increasing intensity (55, 60, 75 and 90% individual AMT). Both sessions consisted of four blocks of different TMS intensities. Each block included 30 trials of the phonological task and was separated by 5 min rest to prevent carry-over effects. The order of sessions was counterbalanced across subjects. In all other aspects, this experiment was identical to the main experiment. Overall RT were again significantly increased for auditorily compared to visually presented words (*F*_1,6_ = 122.93; *p* < 0.0001). A main effect of intensity (*F*_3,18_ = 3.80; *p* = 0.029; Fig. 4A and B) showed that RT were significantly longer with TMS at an intensity of 90% AMT compared to all other intensities (*t*_6_ = 2.44; *p* = 0.02; *t*_6_ = 2.95; *p* = 0.006; *t*_6_ = 2.16; *p* = 0.04 for 90 vs. 55, 60 and 75%; respectively). This intensity effect was comparable for left and right pIFG TMS (*p* = 0.74). ER were not significantly different between the different conditions (all *p* > 0.13; Fig. 4C and D).

### 3.4 Unpleasantness scores

All subjects in the control group rated the three different sham TMS conditions as neutral (1). In the pIFG group, pre-experimental (mean: 1.29, 1.29, 1.36; standard deviation: 0.91, 0.63, 0.66 for left, right and bilateral stimulation, respectively) and post-experimental ratings (mean: 1.21, 1.43, 1.79; standard deviation: 0.58, 0.76, 0.97) were not significantly different. There were also no significant differences between pre-experimental (mean: 1.43, 1.43, 1.71; standard deviation: 0.51, 0.65, 0.61) and post-experimental ratings (mean: 1.71, 1.36, 1.86; standard deviation: 0.91, 0.63, 0.66) in the aIFG group nor between the two groups (i.e. pIFG vs. aIFG).

## 4 Discussion

Using a perturb-and-measure approach, we compared the disruptive effects of high-frequency TMS over the left, right and bilateral posterior and anterior inferior frontal gyri during phonological and semantic word decisions. This allowed us to test three different explanations for why fMRI studies show bilateral pIFG activation during phonological decision tasks but lesion studies emphasize the importance of left but not right hemisphere damage in aphasia (see Section 1 for details). Our finding that reaction times and error rates increased following TMS to right pIFG as well as left pIFG indicates that unperturbed right pIFG activation is necessary for accurate and efficient phonological decisions in the healthy brain. Moreover, our finding that phonological decision performance was not worse for bilateral pIFG TMS than unilateral pIFG TMS provides no evidence that the left and right pIFG can compensate for one another: If phonological decisions are possible with either the left or the right pIFG, then dual-site TMS over the left and right IFG should produce a greater “lesion” effect than TMS over left or right pIFG alone. In contrast, our observation that the behavioural effect of TMS on phonological judgements was the same for unilateral and bilateral TMS suggests that the left and right pIFG are equally necessary for phonological decisions in the healthy brain.

Our finding that online TMS over the right pIFG selectively interfered with phonological but not semantic judgements provides the first strong evidence that right pIFG is necessary for efficient phonological processing in healthy right-handed subjects. The disruptive effect was independent of the presentation modality (i.e. auditory or visual) and was present during unilateral as well as bilateral TMS. Therefore, it cannot be explained in terms of the contralateral hemisphere playing a compensatory role (see Price & Friston, 2002). To the contrary, both the main experiment and the follow-up experiment manipulating TMS intensity indicated that the lesion effect of unilateral TMS to the right pIFG was comparable to the lesion effect induced by unilateral TMS to the left pIFG or bilateral TMS to the right and left pIFG. Moreover, it cannot be explained in terms of a speed-accuracy trade-off because the detrimental effects of right pIFG TMS on reaction times were paralleled by an increase in error rates. Although the TMS-induced change in behavioural measures was stronger for reaction times than error rates, TMS over pIFG but not aIFG increased error rates, especially when given over the right hemisphere. We thus conclude that the right pIFG is necessary for efficient *and* accurate phonological decisions in the healthy brain.

To the authors’ knowledge, no study to date has investigated the effects of TMS to right pIFG during phonological processing although several functional imaging studies revealed bilateral activity in the pIFG when healthy right-handed subjects made phonological decisions (Chee et al., 1999; Devlin et al., 2003; Poldrack et al., 1999). Nevertheless, our results confirm several recent TMS studies demonstrating that the left pIFG is important for phonological processing of visually presented words (Gough et al., 2005; Nixon et al., 2004; Romero et al., 2006). For example, Gough et al. (2005) applied 10 Hz online TMS to either left pIFG or aIFG while right-handed healthy subjects had to decide whether or not two visually presented words sounded the same (phonological task) or meant the same (semantic task). Their results revealed a double dissociation within the left IFG with TMS over left pIFG selectively increasing reaction times for the phonological but not the semantic task and vice versa for left aIFG stimulation. Our results extent these findings by showing that the right pIFG also contributes to phonological processing and that the effect is observed irrespective of whether the stimuli are written words or auditory words.

The finding by Gough et al. (2005) that TMS over aIFG impaired semantic decisions more than phonological decisions contrasts with that of Kohler, Paus, Buckner, and Milner (2004) who found that high-frequency online TMS to the left but not right aIFG enhanced the accuracy of semantic word encoding in comparison to TMS over parietal sites. The authors concluded that TMS to left aIFG might have triggered a more extensive processing of the stimulated items underlining the important role of the left aIFG in episodic memory function.

We did not find a significant influence of aIFG stimulation on semantic processing as implicated by Gough et al. (2005) and Kohler et al. (2004). This is striking since we used a comparable TMS protocol to the Gough et al. study (10 Hz TMS starting 100 ms after word onset). However, in these previous studies, stimulation intensity ranged from 100 to 110% resting motor threshold or 60% total stimulator output compared to 90% active motor threshold (approximately corresponding to 29% total stimulator output) in the present study. We refrained from using higher stimulation intensity because subjects reported substantial discomfort and muscle contractions at stimulus intensities above 100% active motor threshold in a pilot study, especially when TMS was given over the aIFG. It is thus very likely that our stimulation intensity was too low to effectively disrupt semantic processing in the aIFG. However, the low stimulation intensity was sufficient to disrupt phonological processing in the pIFG. These results cannot be attributed to task difficulty since both tasks yielded comparable RT and ER in the control group (sham TMS). One possible explanation is that the semantic network was able to compensate for the disruptive effect of low-intensity TMS over left aIFG.

An alternative interpretation of our results is that TMS over unilateral right pIFG affected phonological processing in the left pIFG by activating transcallosal inputs from the right to the left pIFG (see Siebner, Hartwigsen, Kassuba, & Rothwell, 2009; Thiel, Schumacher, et al., 2006). This interpretation would be in line with previous TMS studies demonstrating acute remote effects of TMS in contralateral homotopic areas (Baumer et al., 2006; Bestmann et al., 2008; Bestmann, Baudewig, Siebner, Rothwell, & Frahm, 2005; Irlbacher, Voss, Meyer, & Rothwell, 2006; Thiel, Schumacher, et al., 2006). For example, it has been shown that TMS over the motor cortex can change the metabolic rate in the contralateral motor areas and may lead to behavioural or functional effects ipsilateral to the side of stimulation (Paus et al., 1998; Siebner et al., 2000; Strafella & Paus, 2001). However, several considerations render this explanation unlikely. Neurophysiological studies of the primary motor cortex showed that TMS over the ipsilateral motor hand area has much stronger excitatory and longer lasting inhibitory effects on regional excitability as opposed to the transcallosally induced effects induced by TMS over the contralateral motor hand area (Kobayashi & Pascual-Leone, 2003). The threshold for inducing transcallosal inhibitory effects is also considerably higher than for inducing intracortical inhibition with the coil placed over the motor cortex (Ferbert et al., 1992; Kujirai et al., 1993). Hence, the effect size of a lesion effect should be stronger and the threshold for inducing a lesion effect should be lower with ipsilateral than contralateral TMS using the same stimulation intensity. This was not the case in the present study. The threshold as well as the magnitude of the disruptive effect on phonological decisions was comparable with TMS to both hemispheres as supported by our follow-up experiment.

Our results significantly extend current neurobiological concepts of the human language system by showing that language processing involves more than a left-hemisphere specialization. This may have implications for the interpretation of functional imaging studies showing right IFG language-related activation in aphasic patients with left-hemisphere damage (Raboyeau et al., 2008; Saur et al., 2006; Winhuisen et al., 2005, 2007). While recent studies indicate that the (temporary) recruitment of homologue areas in the right hemisphere after left-hemisphere stroke may be beneficial, longer term language improvement is associated with left-hemisphere language function (Saur et al., 2006; Thiel, Habedank, et al., 2006; Winhuisen et al., 2005). For example, Winhuisen et al. (2007) argue that restoration of the left-hemisphere network seems to be more effective for recovery after stroke, but in some cases, right-hemisphere areas are integrated successfully. Likewise, Dewarrat et al. (2009) showed that word comprehension and repetition were impaired after right-hemisphere damage but less frequently than after left-hemisphere damage. Our TMS results contribute by showing that the involvement of right-hemisphere language areas is not limited to recovery after stroke but is also essential for phonological processing in healthy subjects.

In contrast to the evidence showing that the right frontal cortex contributes to phonological processing, other studies have shown improved language recovery in aphasic patients following suppression of neuronal processing in the non-lesioned right IFG with transcranial stimulation techniques (Andoh & Martinot, 2008; Martin et al., 2009; Naeser et al., 2005a, 2005b): The behavioural improvement after suppression of neuronal processing in the non-lesioned right IFG has been interpreted as a suppression of maladaptive “over-activation” in the right hemisphere which in turn may have allowed for a better modulation in the remaining left-hemisphere networks (Naeser et al., 2005a). It should be noted, however, that the experimental design of this study was different from ours. Specifically, we applied TMS online (i.e. during task performance), leaving the language system no time to develop adaptive plasticity. In contrast, the above cited studies of stroke patients used a different TMS protocol, where TMS was applied offline (i.e. before the task). Further, while we contrasted phonological with semantic judgements, the above cited studies used picture naming and solely targeted the anterior part of the IFG which is associated with semantic rather than phonological processing (e.g. Devlin et al., 2003; Fiez, 1997; Gitelman et al., 2005; Gough et al., 2005; Poldrack et al., 1999). Together, the current set of results motivates future investigation of the functional relevance of the right pIFG over the course of recovery from left-hemisphere stroke. For example, the right pIFG may be more functionally relevant in the acute phase after stroke than in the chronic phase when reorganisation of the language networks has occurred (Saur et al., 2006).

In a recent study, Raboyeau et al. (2008) investigated the involvement of the right inferior frontal cortex in recovery after left-hemisphere stroke. Using positron emission tomography (PET), the authors found increased activation of the right inferior frontal gyrus in both aphasic patients and healthy subjects during word retrieval following difficult re-learning. Based on this finding, it was concluded that right inferior frontal activations were not a mere consequence of left-hemisphere lesions as they existed in patients as well as healthy subjects who had to work out the phonetic/phonologic forms of once learned but forgotten foreign words. Accordingly, right inferior frontal activation was related to lexical retrieval following re-learning. In their study, right IFG activity increased with performance improvement in aphasic patients. Although this seems to contradict studies suggesting that right-hemisphere activation in chronic aphasics could be deleterious for language recovery when left frontal gyrus is not totally damaged (e.g. Buckner, Corbetta, Schatz, Raichle, & Petersen, 1996; Naeser et al., 2005b; Rosen et al., 2000), Raboyeau et al. (2008) argue that most studies only investigated chronic word retrieval deficit processing which is different to the dynamic lexical learning processes examined in their study. Our results are in good agreement with the findings by Raboyeau et al. (2008) since we also show the contribution of a right inferior frontal region to phonological processing in healthy subjects. Although our syllable judgements are less difficult than the task used by Raboyeau et al. (2008), both require phonological working memory processes. As emphasized by Seghier et al. (2001), further investigations on aphasic patients with right-hemisphere lesions are necessary to understand the large literature on aphasic patients with left-hemisphere damage.

In conclusion, our findings extend current concepts by showing that both, the right and left pIFG, are critical nodes within the neural network implicated in phonological processing. Our study highlights the importance of the right posterior inferior frontal gyrus during phonological decisions in healthy right-handed subjects independent of the modality that the words are presented in. Future studies are now required to systematically investigate the effect of right inferior frontal damage on the efficiency of phonological decisions in patients. According to our results, we would predict that these patients have some degree of phonological processing impairment, irrespective of whether words are presented in the auditory or visual modality.

## Figures and Tables

**Supplementary Fig. I:**
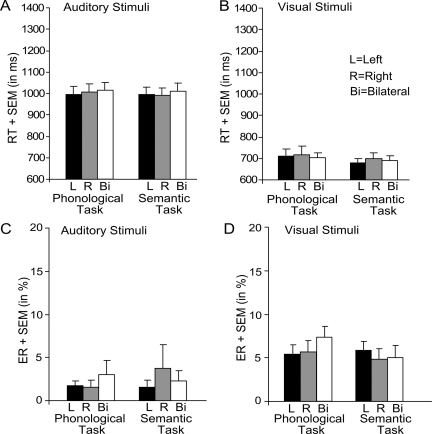
Mean reaction times (RT) are and error rates (ER) for the two different tasks in the sham TMS group. In (A and B), RT are displayed. ER are shown in (C) and (D). For illustrating purposes, responses for auditorily and visually presented words and the different sham TMS sites (left, right, bilateral) are displayed in different panels. Neither RT nor ER were significantly different between the phonological and the semantic task. Error bars represent onefold standard error from the mean (SEM); ms = milliseconds.

**Table 1:** Reaction times and error rates for the different tasks in both groups.

	Auditory word stimuli		Visual word stimuli	
	RT ± SEM (ms)	ER ± SEM (%)	RT ± SEM (ms)	ER ± SEM (%)
Group receiving TMS over the pIFG (*n* = 14)				
Task: phonological word judgement				
TMS to left pIFG	1157 ± 57.51	0.12 ± 0.04	792 ± 53.03	0.09 ± 0.02
TMS to right pIFG	1166 ± 56.78	0.12 ± 0.04	792 ± 58.98	0.10 ± 0.02
Bilateral TMS of pIFG	1176 ± 59.07	0.12 ± 0.04	802 ± 57.34	0.07 ± 0.02

Task: semantic word judgement				
TMS to left pIFG	978 ± 41.25	0.08 ± 0.03	712 ± 38.62	0.03 ± 0.01
TMS to right pIFG	977 ± 33.78	0.06 ± 0.03	714 ± 32.50	0.04 ± 0.01
Bilateral TMS of pIFG	970 ± 39.26	0.07 ± 0.04	723 ± 33.15	0.03 ± 0.01

Group receiving TMS over the aIFG (*n* = 14)				
Task: phonological word judgement				
TMS to left aIFG	1031 ± 37.13	0.02 ± 0.01	676 ± 15.78	0.07 ± 0.02
TMS to right aIFG	1029 ± 45.17	0.02 ± 0.01	665 ± 18.22	0.06 ± 0.02
Bilateral TMS of aIFG	1029 ± 43.54	0.04 ± 0.01	688 ± 21.70	0.05 ± 0.02

Task: semantic word judgement				
TMS to left aIFG	1013 ± 28.30	0.04 ± 0.01	687 ± 10.72	0.06 ± 0.03
TMS to right aIFG	1016 ± 28.97	0.06 ± 0.02	690 ± 13.12	0.05 ± 0.02
Bilateral TMS of aIFG	1017 ± 32.82	0.04 ± 0.01	710 ± 15.44	0.05 ± 0.02

ER = error rates; RT = reaction times; SEM = standard error of the mean (in milliseconds or percent of all trials).
